# Study on the Physical Properties of a SiNW Biosensor to the Sensitivity of DNA Detection

**DOI:** 10.3390/ma14195716

**Published:** 2021-09-30

**Authors:** Siti Noorhaniah Yusoh, Khatijah Aisha Yaacob

**Affiliations:** Engineering Campus, School of Materials & Mineral Resources Engineering, Universiti Sains Malaysia, Nibong Tebal 14300, Pulau Pinang, Malaysia; haniah.yusoh@gmail.com

**Keywords:** silicon nanowires, dengue biosensor, sensitivity, dimension, number of wires, surface roughness

## Abstract

SiNW (silicon nanowire) arrays consisting of 5- and 10-wires were fabricated by using an atomic force microscope—the local anodic oxidation (AFM-LAO) technique followed by wet chemical etching. Tetramethylammonium hydroxide (TMAH) and isopropyl alcohol (IPA) at various concentrations were used to etch SiNWs. The SiNWs produced were differed in dimension and surface roughness. The SiNWs were functionalized and used for the detection of deoxyribonucleic acid (DNA) dengue (DEN-1). SiNW-based biosensors show sensitive detection of dengue DNA due to certain factors. The physical properties of SiNWs, such as the number of wires, the dimensions of wires, and surface roughness, were found to influence the sensitivity of the biosensor device. The SiNW biosensor device with 10 wires, a larger surface-to-volume ratio, and a rough surface is the most sensitive device, with a 1.93 fM limit of detection (LOD).

## 1. Introduction

Silicon nanowires (SiNWs) are categorized as one-dimensional (1D) nanomaterials with diameters in the range of 2 to 100 nm, exhibiting two types of conductivity—p-type and n-type conductivity [1,2,3]. Silicon nanowires can be fabricated by using two approaches—bottom-up or top-down—with controllable compositions, morphologies, structures, and dimensions [1,4,5]. Nanotechnology has discovered that SiNWs have great potential in being used as biosensor transducers for biomolecule detection, e.g., enzymes, antibodies, antigens, RNA, and DNA [6,7,8]. The SiNWs are widely used in biosensor research, since they are highly sensitive devices [9,10,11]. SiNWs are attracting attention because of their exclusive properties, which can be integrated into biosensor device structures [12].

A biosensor is a device that has the ability to detect a biomolecule-related element with a suitable transducer and translate it to a measurable signal [13]. A biological signal is detected and transduced by electrical, electrochemical, physical, chemical, optical, and thermal properties into observable information, which is analyzed quantitatively [14]. Most transducers are nanostructures, such as nanowires, nanotubes, nanorods, and nanoparticles. The nanostructure is a crucial element for future biosensor devices due to its small size. Innovations in transducers have conceivably produced useful approaches to new detection techniques, transforming the way in ‘sensing’.

In recent decades, SiNWs have become potential transducer elements used to enhance the sensitivity of biosensors. Biosensor device-based SiNW field-effect transistors (SiNWs-FETs) have been developed, originating from Piet Bergveld, who developed the ion sensitive field-effect transistor (ISFET) in 1970 [15]. Efficient performance of a SiNW biosensor can be determined by many factors, such as orientation of the silicon substrate, dopant density, the number of SiNWs, and the diameter of SiNWs [4,16]. A previous study found the 20 nm of SiNWs sensitively detected dengue virus DNA at 2 fM [17]. The small diameter of SiNWs could give a larger surface-to-volume ratio and increase the sensitivity. In this paper, physical factors of SiNWs, such as the number of wires, dimensions of the wires, and the surface roughness that affects the sensitivity of biosensors, are studied and discussed.

## 2. Materials and Methods

### 2.1. Materials and Reagents

The p-type silicon-on-insulator (SOI) wafer was chosen as a substrate to fabricate a SiNW biosensor device. Chemicals, such as tetramethylammonium hydroxide (TMAH) and isopropyl alcohol (IPA) were purchased from Sigma Aldrich and were used for wet chemical etching. Furthermore, 3-aminopropyl triethoxysilane (APTES), glutaraldehyde, and phosphate buffered saline (PBS) at pH 7.4 were used for SiNWs surface modification. Moreover, 23-mer of peptide nucleic acid, PNA (N-GCTCCTTCTTGTGATCCTAGTAC-C) was purchased from Panagene and was used as a sensor probe. In addition, 23-mer of DEN-1 (5-GTACTAGGATCACAAGAAGGAGC-3) in single stranded deoxyribonucleic acid (ssDNA) form was used. The DNA oligomers were purchased from First Base Laboratories Sdn Bhd and was prepared in Tris-EDTA buffer solution (pH 8.0).

### 2.2. Fabrication of a SiNW Biosensor Device

The silicon dioxide (SiO_2_) patterns were fabricated by the AFM-LAO technique using a scanning probe microscope (SPI3800N). The parameters for fabrication, such as a cantilever-coated tip (Au), applied voltage (9 V), writing speed (0.4 µm/s), relative humidity (55–65%), and temperature (24–26 °C) were applied. The SiO_2_ patterns acted as a mask during the etching process. The unmasked silicon layers were etched by using TMAH (25 wt.%) added with IPA (10, 20, and 30 vol.%) for 30 s. Then, the SiO_2_ layer was removed using hydrofluoric acid (HF), diluted with deionized water (DIW), with a ratio of 1:100 for 10 s immersion to obtain the final device structure.

### 2.3. Functionalization of the SiNW Surface

The SiNW surface was functionalized by using APTES and glutaraldehyde. The APTES at 99% purity was used to immobilize the PNA. The SiNW devices were immersed in 2% of APTES, 5% of deionized water, and 95% ethanol at room temperature for 2 h to allow the hydrolysable group (-OCH_2_CH)_3_ of APTES to adhere to the surface of the SiNWs. The unreacted APTES was then washed off by rinsing the SiNWs device with ethanol.

Glutaraldehyde was used as a bifunctional linker to create two aldehyde ends, one of which would bind to the amine-terminated APTES and the other end was free to immobilize the PNA. The SiNW device was immersed into a solution of 2.5% glutaraldehyde solution with phosphate buffered saline (PBS) for 1 h and unbounded glutaraldehyde was washed with PBS solution.

In this study, 10 µM PNA was prepared and injected onto the surface of the SiNWs for immobilization, followed by an overnight incubation process. The PNA functionalized SiNWs were then hybridized with DEN-1 at different concentrations: 1 fM, 10 fM, 1 pM, 10 pM, 1 nM, 10 nM, 1 µM, and 10 µM. DEN-1 was injected onto PNA-functionalized SiNWs and incubated for 2 h at room temperature prior to electrical measurement.

### 2.4. Characterization of a SiNW Biosensor Device for DNA Detection

Fabricated SiNWs were physically characterized using AFM topography to study dimension and roughness. The functionalized SiNW biosensor devices were subjected to electrical characterization to investigate the current–voltage (I–V) properties using semiconductor parameter analysis (SPA, Agilent, HP4156C, Rocklin, CA, USA) with a Lakeshore probing system. Specificity, sensitivity, and limit of detection (LOD) were calculated from the I–V results obtained.

The current–voltage (I–V) measurements of SiNW biosensor devices were measured at room temperature. The I–V was measured by probing two terminals of the source (S) and the drain (D) pad as cathode and anode probes, respectively. A sweep drain–source voltage (V_ds_) of 0 to 1 V was supplied to the SiNW device, allowing the current (I_ds_) to flow from the drain to the source, creating an I–V curve. Sensitivity and limit of detection (LOD) of all devices were calculated from the I–V measurements obtained.

## 3. Results

### 3.1. Fabrication of the SiNW Biosensor Device

**Figure 1:**
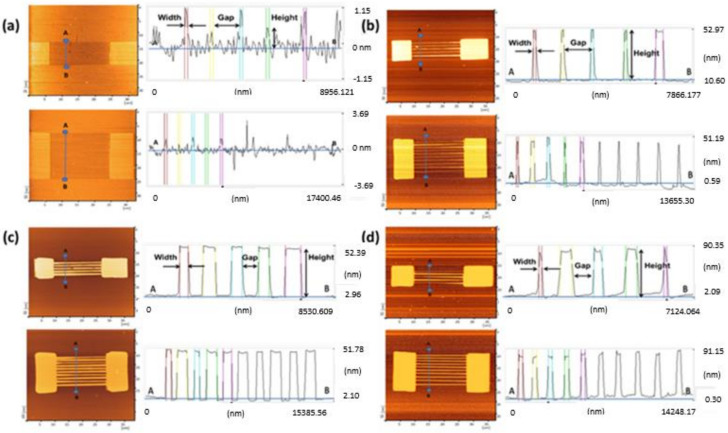
(**a**) AFM profile image of 5 wires and 10 wires before etching. AFM profile image of 5-wire and 10-wire SiNW devices after etching with addition of (**b**) 10 vol.%, (**c**) 20 vol.%, (**d**) 30 vol.% IPA.

**Figure 2:**
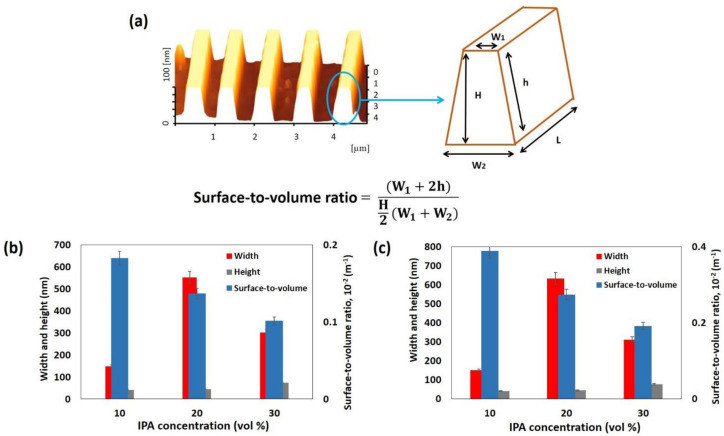
(**a**) Schematic etching profiles; H represents height, W_1_ and W_2_ are widths, h is hypotenuse, and L is length. The width, height, and surface-to-volume ratio for (**b**) 5-wire (**c**) and 10-wire devices.

**Figure 3:**
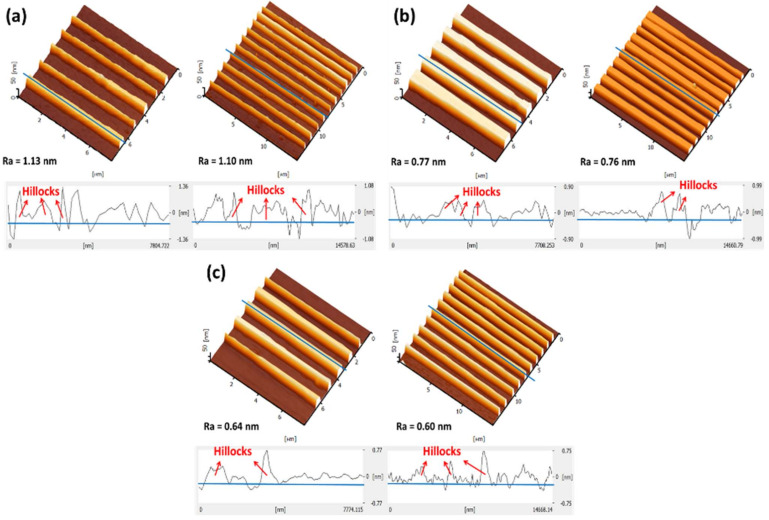
Surface roughness (Ra) of 5-wire and 10-wire devices after etching with TMAH 25 wt.% and IPA, (**a**) 10 vol.%, (**b**) 20 vol.%, (**c**) 30 vol.%.

Two types of SiO_2_ patterns, consisting of 5- and 10-wire arrays, were fabricated via the AFM-LAO technique, as depicted in Figure 1a. The patterns were etched using TMAH 25 wt.% with different additions of IPA concentrations: 10, 20, and 30 vol.% at 65 °C for 30 s. The fabricated SiNW devices were characterized under AFM contact mode topography and the results shown in Figure 1b–d were obtained with respect to the etching parameters. All SiNWs produced have a trapezoidal shape [8]. The width and height of the SiNWs were measured directly with the AFM software. By using the widths and heights of the produced SiNWs after etching, the surface-to-volume ratio for all SiNWs were calculated using the formula shown in Figure 2a. Figure 2b,c, display the results of the width, height, and surface-to-volume ratio of SiNWs etched with 10, 20, and 30 vol.% of IPA. The widths and heights of SiNWs etched with different IPA concentrations represent different values for both 5-wire and 10-wire devices. This is because a different concentration of IPA gives a different etching rate that influences the amount of silicon layer removal, which contributes to a different value of the surface-to volume ratio. In the etching solution, the Si surface was found to be ‘hydrogen-terminated’ and the surface became hydrophobic. The hydrocarbon chains from IPA molecules bound to the hydrogen-terminated Si surface by van der Waals forces. As the IPA concentration increased, the IPA and TMAH solutions became saturated, causing the disturbance of the hydrogen-bonding network between the IPA molecules and the hydrogen-terminated silicon surface with more IPA molecules, which caused an increase in the etching rate [18]. A fast etching rate removes more uncovered silicon layers and produces higher or thicker SiNWs. Still, surface roughness (Ra) of SiNWs was also affected when different concentrations of IPA were used during the etching process. Figure 3 indicates the roughness of SiNW devices that were etched at concentrations of 10, 20, and 30 vol.% of IPA. The results show that the surface roughness decreases as the IPA concentration increases. This shows that the addition of IPA concentration improves the surface roughness of the SiNWs.

### 3.2. Electrical Characteristic of a SiNW Biosensor Device

**Figure 4:**
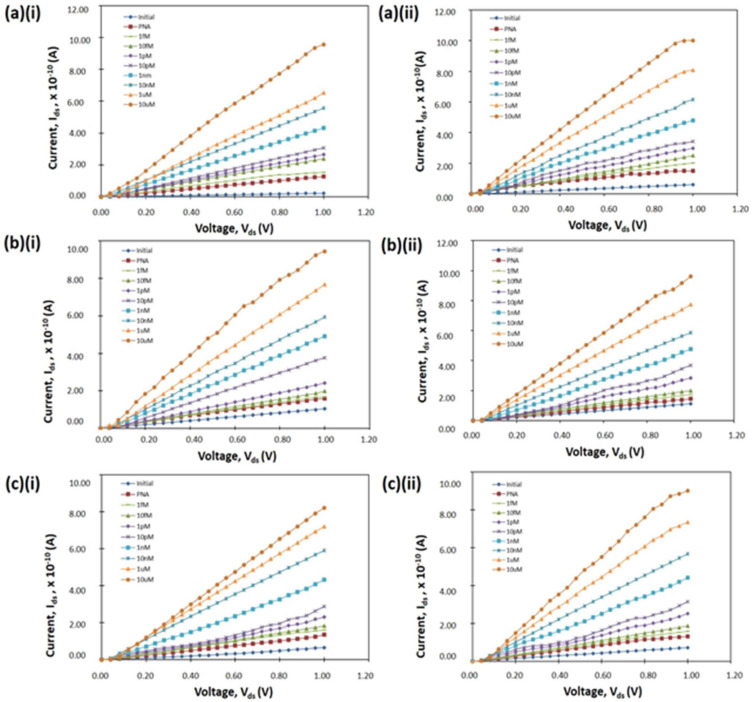
Current–voltage curves of SiNW devices etched using IPA at various concentration; (**a**) 10 vol.%, (**b**) 20 vol.%, (**c**) 30 vol.% for (**i**) 5-wires and (**ii**) 10-wires.

**Figure 5:**
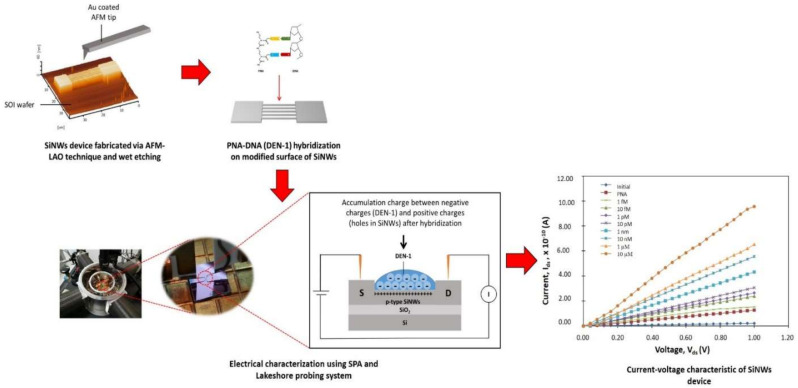
Electrical characterization of the SiNW devices using SPA and the Lakeshore probing system. The accumulation of negative charges from DEN-1 and positive charges of SiNWs occurred after hybridization and resulted in changes in resistance that caused the current to increase.

The SiNW devices have different properties with respect to the number of wires, dimensions, and surface roughness. The differences resulted in various electrical characteristics of the devices. The I–V curves for all devices are shown in Figure 4. The I–V results show that the current flow through the device increased with increasing DEN-1 concentration. This was due to the increased concentration in DEN-1, which induced a more negative charge of the DEN-1 phosphate backbone and promoted more positive charge carriers (holes) that accumulated in the p-type SiNW device (Figure 5). Therefore, more changes in resistance cause an increase in the current. This result corresponds with results by Zhang et al. (2010), who demonstrated that, with the increase in the concentration of the target analyte, more changes were presented, which influenced the reaction of the biosensor.

**Figure 6:**
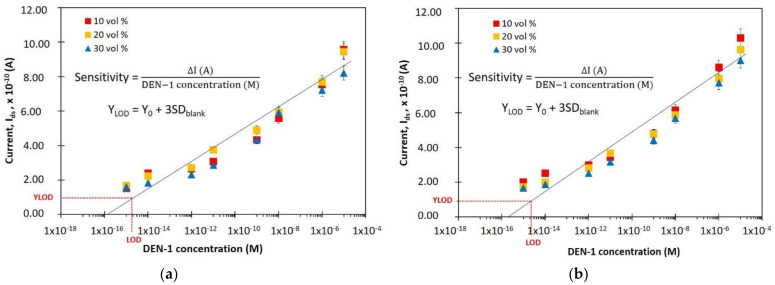
Sensitivity and LOD of SiNWs for (**a**) 5-wire (**b**) and 10-wires device.

The biosensor performance in detecting the target analyte can be described by sensitivity. To exhibit the sensitivity of the device, the effect of DEN-1 concentration in the range of 1 fM to 10 µM on the current (I_ds_) were measured at 1 V (V_ds_). The I_ds_ calibration curves relative to DEN-1 concentration were plotted as shown in Figure 6, with the slope of the calibration curve equal to the sensitivity of the device. Sensitivity is also defined as the slope of the calibration curve, the value of I_ds_ (A) gradient over the DEN-1 concentration (M) gradient. The results of sensitivities for all devices are shown in Table 1, which shows that the 10-wire device was etched with 25 wt.% of TMAH and 10 vol.% of IPA has the highest sensitivity (82.8 µAM^−1^).

Moreover, the performance of the biosensor device can also be evaluated by calculating the limit of detection (LOD). The LOD of the biosensor device is the smallest concentration detectable by the particular device. The procedure for calculating LOD is to plot a curve of the calculated relative change in the current at the various concentrations of DEN-1, ranging from 1 fM to 10 µM. Then Y_LOD_ is calculated using Y_LOD_ = Y_(baseline, immobilization)_ + 3σ [17,19]. The Y_(baseline, immobilization)_ is the response of the blank signal and is assumed to be 0, while σ is the standard deviation of the regression calibration curve [20,21,22]. The calculated Y_LOD_ in the calibration curve extends to X_LOD_, which corresponds to the lowest detectable DEN-1 concentration. Table 1 summarizes the LODs for each device. The sensitivity and LOD for each device has a different value due to their physical factors, the number of wires, the dimensions of the wires, and the surface roughness.

### 3.3. Factors Affecting Sensitivity a SiNW Biosensor

#### 3.3.1. Number of Wires

**Figure 7:**
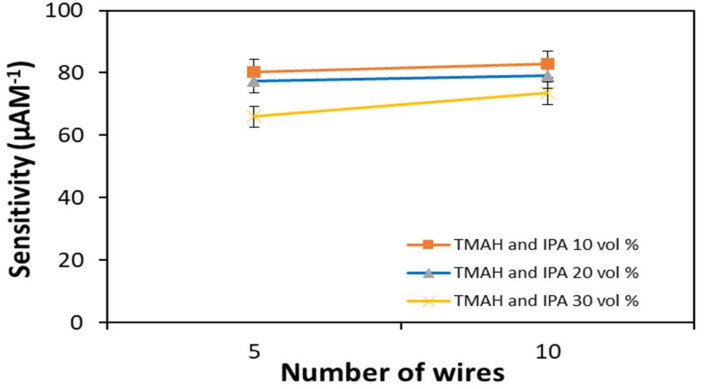
Sensitivity of devices relative to number of wires.

The number of wires is believed to be one of the parameters that affects the sensitivity of biosensors. A previous study reported that the number of bridging nanowires fabricated in a device is an important factor in determining sensitivity [22]. In this study, for a 5-wire device, an initial source–drain current of I_0(5w)_ was experienced; introducing the DEN-1 analyte resulted in the hybridization of DEN-1 onto the SiNW surface, to produce a change in the device current (I − I_0_)_5w_. The sensitivity was calculated by (I − I_0_)_5w/_DEN-1 concentration. Similar to the 10-wire device with an initial source-drain current of I_0(10w)_, the sensitivity of the 10-wire device was calculated as (I − I_0_)_10w_/DEN-1 concentration. Figure 7 indicates the plot of sensitivity against the number of wires consisting in the SiNW devices. The results show higher sensitivity if the device has more wires, irrespective of etching parameters. The increase in sensitivity of the devices etched with 10% by volume IPA and 20% by volume IPA is small compared to the device etched with 30% by volume IPA.

This finding is different from the work conducted by Li and co-workers. They suggested that sensitivity decreased with an increased number of wires [16]. They claimed that a device consisting of a single wire was more sensitive compared to a device with 4 to 7 wires. The difference might be because the structures of SiNWs fabricated in this study were horizontally on the p-type silicon substrate via the AFM lithography technique. In contrast, Li et al. (2011) grew the removable n-type silicon nanowires, bridging between the source and drain of aluminum electrodes, using the chemical vapor deposition (CVD) technique.

#### 3.3.2. Dimension of Wires

**Figure 8:**
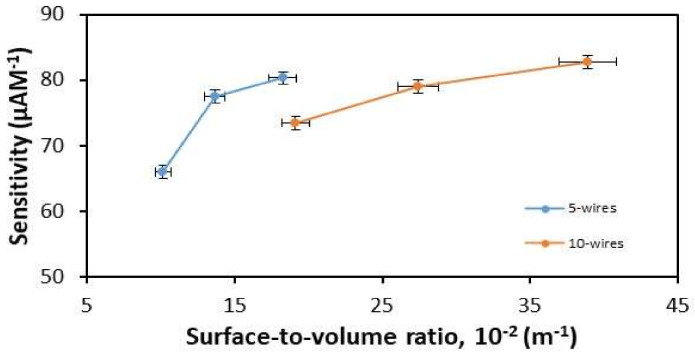
Sensitivity vs. surface-to-volume for 5-wire and 10-wire devices.

The SiNWs produced after etching had different width and height dimensions, which influenced the surface-to-volume ratio. In this study, it was found that SiNWs with smaller width and height contributed to a larger ratio of surface-to-volume, while the larger width and height had a smaller ratio surface-to-volume. The sensitivities of the devices were investigated at different surface-to-volume ratios. Figure 8 shows the sensitivities of devices with increasing surface-to-volume ratio for 5-wire and 10-wire devices. The results obtained show that sensitivity increases as the surface-to-volume ratio increases. This is due to the fact that the larger surface-to-volume ratio has a smaller cross-section, which means that the hole charge carriers can easily move along the wire. This makes it more sensitive and leads to large changes in the current and conductance.

#### 3.3.3. Surface Roughness

**Figure 9:**
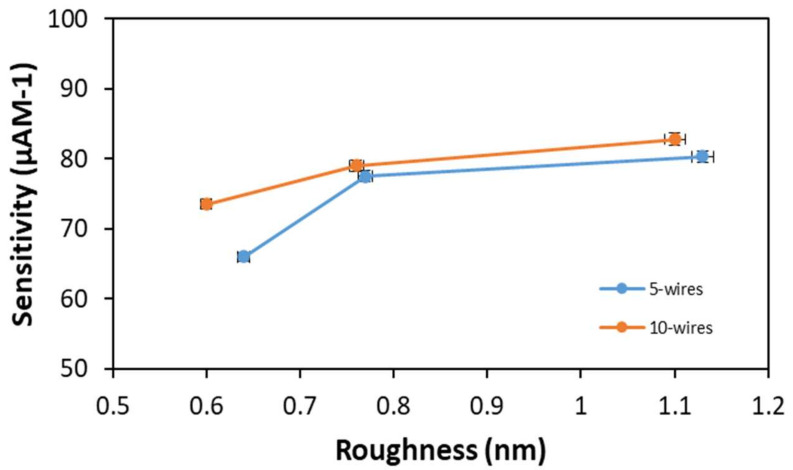
The 5-wire and 10-wire device sensitivity vs. surface roughness.

The surfaces roughness of wires after etching varied for each case because of the usage of different IPA concentrations during the wet chemical etching process. Figure 9 indicates the relation between sensitivity and surface roughness of SiNWs for 5-wire and 10-wire devices. It shows the sensitivity for both devices increasing as the roughness of SiNWs increases for 5-wire and 10-wire devices. This shows that physical factors, such as surface roughness, could affect the sensitivity of a biosensor device. Additionally, a rough surface contributes to a large surface-to-volume ratio, which could improve device performance [23].

### 3.4. Specificity of a SiNW Biosensor

**Figure 10:**
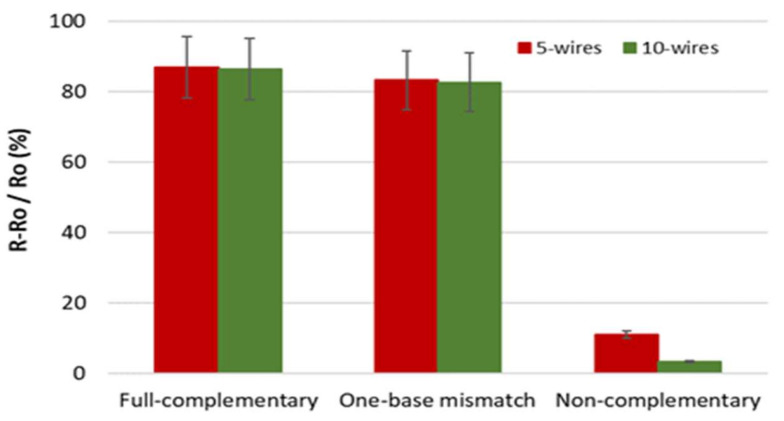
Hybridization specificity (devices etched with TMAH and IPA 10 vol.%) presented by changes in resistance to full-complementary, one-base mismatch, and non-complementary sequences.

The applicability and selectivity of the biosensor are shown in terms of specificity. Device specificity was studied and presented as the percentage change in resistance among PNA immobilized with full-complementary, one-base mismatch, and non-complementary sequences. In this study, 5- and 10-wire devices etched with TMAH and IPA 10 vol.% were found to have the lowest LODs compared to others. The specificities of both devices were studied, and are shown in Figure 10. The 5-wire device has a percentage of 87%, 83%, and 11% for each sequence, respectively. On the other hand, the 10-wire device has a resistance change of 86%, 83%, and 3% for each hybridization. The results show a significant difference in resistance change between full-complementary, one-base mismatch, and non-complementary sequences. This indicates that the SiNW devices had good specificity in detecting DNA.

## 4. Conclusions

Dengue DNA detection with SiNW devices was carried out successfully because the resistance changes were obtained during I–V measurement. Sensitivity and LOD of the devices were calculated from the I–V measurement. The results show that a 10-wire SiNW device, which was etched using 25% by weight of TMAH, added with 10% by volume of IPA, had the highest sensitivity of 82.8 µAM^−1^, with the lowest LOD at 1.93 fM. The study showed that the number of wires, the dimension of wires, and the surface roughness are physical factors that affect the sensitivity of a SiNW biosensor device for DNA detection.

## Figures and Tables

**Table 1:** The sensitivity and LOD for 5 wires and 10 wires etched with various concentrations of IPA.

Etchants	Devices	Sensitivity (µAM^−1^)	LOD (fM)
TMAH and IPA 10 vol.%	5-wires	80.3	2.22
	10-wires	82.8	1.93
TMAH and IPA 20 vol.%	5-wires	77.5	3.63
	10-wires	79.0	3.20
TMAH and IPA 30 vol.%	5-wires	66.0	4.15
	10-wires	73.5	3.87

## Data Availability

Not applicable.
